# Lipoprotein binding preference of CD36 is altered by filipin treatment

**DOI:** 10.1186/1476-511X-7-23

**Published:** 2008-06-26

**Authors:** Jianshe Zhang, Wuying Chu, Ian Crandall

**Affiliations:** 1 Department of Bioengineering and Environmental Science, Changsha University, Changsha, Hunan, PR China; 2 Department of Medicine, University of Toronto, Toronto, M5S 1A8, Canada

## Abstract

The class B scavenger receptor CD36 binds multiple ligands, including oxidized and native lipoprotein species. CD36 and the related receptor SR-B1 have been localized to caveolae, domains that participate in cell signaling, transcytosis, and regulation of cellular cholesterol homeostasis. Previous work has indicated that the ligand preference of CD36 may depend on the cell type in which it is expressed. To determine if the presence or absence of caveolae is the determining factor for lipoprotein preference, we treated CHO-CD36 and C32 cells with filipin. Filipin treatment rapidly increased the binding capacity of CD36 for the native lipoproteins HDL and LDL, but did not affect the binding capacity of CD36 for oxidized LDL. Filipin treatment affected the distribution of caveolin and CD36 suggesting that the presence caveolae may modulate the ligand preference of CD36. However, its molecular mechanism how CD36 and caveolin interaction in regulating lipoprotein transport remains to be further studied.

## Background

Plasma proteins mediate the transport and delivery of lipids. Alterations of the lipoprotein profile present in serum are associated with the incidence of atherosclerosis. In particular, increased production of the oxidized form of the Low Density Lipoprotein (OxLDL), is thought to precede the appearance of the arterial plaques associated with atherosclerosis [1-3]. Uptake of OxLDL is enhanced in the macrophages present in an arterial plaque and can result in cholesterol accumulation and the formation of foam cells, a process that is thought to be a key event in the development of atherosclerosis [3-5], while oxLDL has been directly implicated in human disease, an understanding of the cellular and molecular mechanisms that control the uptake and efflux of all the lipoprotein classes and their oxidized products may be central to understanding cholesterol-related diseases.

Several cell surface glycoproteins, including SR-A, MARCO, CD68, CD36 and SR-B1 are designated as scavenger receptors and contribute to the uptake of modified lipoproteins [6-11]. CD36, an 88 kDa membrane glycoprotein, is found in several cell types, including platelets, monocytes, macrophages and endothelial cells [12,13], CD36 has been reported to be a multifunctional receptor and it recognizes a wide variety of ligands including OxLDL [5,10], thrombospondin [14,15], collagen [16,17], apoptotic neutrophils [18,19], *Plasmodium falciparum*-infected erythrocytes [20,21] and anionic phospholipids [22,23]. Further studies demonstrated that CD36 expressed in COS 7 or Sf9 cells functioned as a high affinity receptor not only for OxLDL, but also for HDL, LDL and VLDL [8,24]. Several regions of CD36 have been implicated as binding domains for its different ligands, including amino acids 28–93 as the OxLDL binding domain [25], and amino acids 93–120 as the thrombospondin binding region [26].

There is increasing evidence that scavenger receptors play a role in the trafficking of both native and oxidized lipoproteins and that the receptor's membrane microenvironment may play a critical role in its function [27,28] Caveolae are glycosphingolipid and cholesterol-enriched microdomains that contain the scaffolding protein caveolin, receptors, and signaling proteins [29,30]. Such membrane microdomains have been implicated in cellular processes such as membrane protein sorting, signal transduction, receptor activation reviewed in [31] and more recently in cholesterol homeostasis [32] We have previously demonstrated that the ability of the native lipoproteins HDL and LDL, which are responsible for cholesterol efflux and influx respectively, to inhibit the binding of pRBCs to human CD36 is dependent on the cell type in which the receptor was expressed [33,34]. In order to test the hypothesis that differential CD36 ligand preference is a result of the receptor's membrane microenvironment, CD36 expressed in sf9 cells (Sf9-CD36), CD36 stably transfected into CHO cells (CHO-CD36), and CD36 endogenously expressed by C32 cells was assayed for its interactions with HDL, LDL and OxLDL. We observed that all three lipoproteins could bind to CD36 expressed in Sf9 cells, however only OxLDL bound to CHO-CD36 and C32 cells. Treatment of CHO-CD36 and C32 cells with filipin, an agent that disrupts caveolae, caused the lipoprotein binding profile of C32 and CHO-CD36 cells to change to that seen in Sf9-CD36 cells. These findings suggest that the binding of native HDL and LDL to CD36 expressed in CHO or C32 cells is normally restricted and HDL and LDL only interact with CD36 when it leaves the environment present in caveolae and enters the general membrane fraction.

## Materials and methods

### Chemicals and reagents

Grace's insect medium, RPM1 1640, fetal calf serum, geneticin, and trypsin-EDTA were purchased from Gibco BRL (Burlington, ON). Filipin was purchased from Sigma (St. Louis, MO). The anti-CD36 antibody, mAB FA6-152, was purchased from Immunotech, (Westbrook, ME) and a polyclonal anti-caveolin antibody was obtained from Transduction Laboratories (BD Biosciences, Mississauga, ON).

### Cell lines and maintenance

Baculovirus-induced expression of CD36 in Sf9 cells was as described by Guy et al. [33]. Briefly, a baculovirus containing the human CD36 (hCD36) gene was constructed using the BacPAK/9 system following the manufacturer's instructions. The hCD36 construct was modified prior to generating the recombinant viral expression vector by reducing the length of the 5' terminus and introducing a *Xho1 *restriction suite upstream of the start cordon. These modifications were made by PCR with the primers 5'-ACATTGCTCGAGATGGGCTGTGAC CGGA-3' and 5'-GCAAAGGCCTTGGATGG-3'. The purified 900 bp nucleotide fragment was subcloned into pcDNA3 containing the 762 bp nucleotide fragment 3' of the *Stu1 *site in hCD36. The modified 1662 bp hCD36 construct was then subcloned into pBacPAK/9 vector digested by *Xho1 *and *Stu1*. CHO-CD36 cells stably transfected with hCD36 or the vector alone (CHO-mock) were maintained in RPMI 1640 supplemented with 10% fetal calf serum, HEPES (6 g/L), sodium bicarbonate (1.8 g/L) and geneticin (50 mg/L). Sf9 cells were maintained in Grace's Insect Medium (Gibco) supplemented with 10% FBS and penicillin/streptomycin, glutamine and kanamycin. C32 cells were grown in RPMI 1640 supplemented with 10% fetal calf serum, HEPES (6 g/L), sodium bicarbonate (1.8 g/L) and gentamicin.

### Preparation of lipoprotein and Dil-labelling

Human HDL, LDL, and oxLDL were prepared by differential density centrifugation as previously described [33]. Labeling of lipoproteins with the fluorescence probe 1,1' diotsdecyl-3-3-3'-3'-tetramethylindocarbocyanine perchlorate (Dil) was carried out according to previous studies [33,34].

### Dil-lipoprotein binding assay

Binding of Dil-labeled lipoprotein to cells was performed using the method of Calvo et al.[24] with minor modification. CHO-CD36, CHO-mock and C32 cells were grown in 6 or 12 well plates. Prior to staining, cells were released from their wells by treatment with trypsin-EDTA (Gibco BRL) and were collected by centrifugation before being incubated with DiI-labeled lipoproteins at 5–10 μg/ml in PBS containing 1 mM CaCl_2 _and 1 mM MgCl_2 _at 37°C for 1 – 2 hrs. Sf9 cells were cultured in 6-well plates (Corning, NY) before being incubated with Dil-labeling lipoproteins at 10 μg/ml at 20°C for 1.5 hrs. After incubation with Dil-labeling lipoproteins, cells were washed with cold PBS and then fixed with 4% paraformaldehyde in PBS for 20 min at room temperature. Stained cells were then observed with an epi-fluorescence microscope or subjected to flow cytometry.

### Purification of caveolin-enriched membrane fractions

Purification of caveolin-enriched membrane fractions from CHO-CD36 cells was performed as described by Smart et al [46] with some modifications. Ten T-75 flasks (Corning, NY) of confluent CHO-CD36 cells were collected by trypsin-EDTA treatment and centrifugation. The cells were then resuspended with 1 ml of Buffer A (0.25 M sucrose, 1 mM EDTA, 20 mM Tricine, pH7.8). The cell suspension was homogenized twice for 30s with a Ultra-Turrax T8 homogenizer (IKA Labortechnik) and the suspension was transferred into to a 1.5 ml tube and centrifuged at 1,000 ×g for 10 min. The resulting supernatant, designated post nuclear supernatant (PNS), was layered on top of 20 ml of 30% Percoll in Buffer A, and was then centrifuged at 84,000 × g for 30 min in a Beckman Ti70 rotor. The membrane fraction, a visible band about 5 – 7 cm from the bottom of centrifuge tube, was collected and the volume was then adjusted to 2 ml with Buffer A, prior to being placed in a Sorvall TH64 centrifuge tube in ice. The sample was sonicated twice using an Aquasonic Model 150T sonicator (PolyScience). A linear 20% – 10% Optiprep gradient (prepared by diluting Buffer C: 50% Optiprep in 0.25 M sucrose, 6 mM EDTA and 120 mM tricine, pH.7.8) was layered on the top of the sample and then the tube was centrifuged at 52,000 ×g for 90 min in a Sorval TH641 rotor. The top 5 ml of the gradient was collected, designated as the Optiprep fraction, and placed in a fresh TH641 centrifuge tube, and mixed with 4 ml of Buffer C. The sample was then overlaid with 2 ml of 5% Optiprep (prepared by diluting Buffer C with Buffer A) and centrifuged at 52,000 ×g for 90 min at 4°C. After centrifugation a distinct opaque band was present in the 5% Optiprep overlay about 4 – 5 mm above the interface and was collected and designated as the caveolin-rich membrane fraction.

### SDS-PAGE and Western blot analysis of CD36 and caveolin

CHO-CD36 and C32 cells, either untreated or treated with filipin, were collected by trypsin-EDTA treatment and were re-suspended in lysate buffer (50 mM phosphate buffered saline, pH 7.4 (PBS) plus 2 mM EDTA and 1% β-mercaptoethanol) prior to being homogenized. The cell lysate was centrifuged for 15 min at 14,000 g at 4°C, the amount of protein present was determined, and the supernatant was used for SDS PAGE and Western blot analysis. The samples were loaded on a 10% SDS-polyacrylamide gel, separated, and transferred onto a nylon-enhanced nitrocellulose membrane (MSI, Westborough, MA). The membrane was blocked with a solution of PBS plus 0.1% Tween-20 (PBST) containing 5% fat-free milk (w/v) for 2 hours at room temperature or overnight at 4°C. A monoclonal anti-human CD36 antibody FA6-152 (Immunotech) or a polyclonal rabbit anti-caveolin antibody (Transduction Laboratories) was used at a concentration of 1:1000 diluted with the blocking solution at room temperature for 4 hours or at 4°C overnight. The presence of the primary antibodies on the membranes was detected using either anti-mouse IgG conjugated to HRP, or anti-rabbit IgG conjugated to HRP (Biolabs, Surrey, BC) at a dilution of 1:2,500 in PBST with 1% (w/v) fat-free milk for 1 hour at room temperature. After washing the blot three times for 10 min each with PBST, the blots were incubated for 1 min in a mixture of equal volumes of LumGLO Chemiluminescent Substrates 1 and 2 (Kirkegaard & Perry Laboratories, Gaithersburg, MD) before exposure to Kodak X-Omat film.

### Determination of CD36 cell surface levels by flow cytometry and immunofluorescent staining

Determination of CD36 surface protein levels on CHO-CD36, CHO-mock or C32 cells was carried out according to [27]. Briefly, treated cells were collected and dispensed into a series of tubes. The cells were washed three times with PBS plus 5% mouse serum for 30 min to reduce non-specific Ig absorption. The cells were then incubated with FITC-conjugated anti-human CD36 antibody at a concentration of 1:200 in PBS for 2 hours at room temperature. After washing 3 times with PBS, 30 μl of the sample was removed for examination using an epifluorescent microscope (Nikon) and the remaining portion of the sample was fixed with 4% paraformaldehyde and analyzed by flow cytometry.

## Results

### Dil-lipoprotein binding in three cell lines, Sf9-CD36, CHO-CD36 and C32

**Figure 1:**
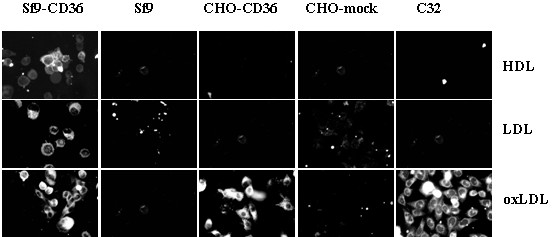
**Comparison of lipoprotein binding to CD36 expressed in three cell lines**. Sf9-CD36, Sf9, CHO-CD36, CHO-mock and C32 cells were grown on glass coverslips and incubated with Dil-labeled lipoproteins (as indicated) at 10 ug/ml of lipoprotein in culture medium at 37°C for 2 – 4 hours. Dil-labeled lipoproteins was determined by examining the cell layers using a fluorescence microscope. Typical images were recorded.

**Figure 2:**
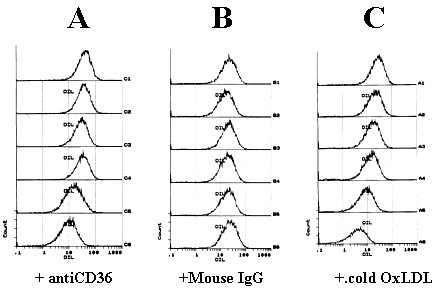
**Effect of specific antibodies against human CD36 and unlabeled oxLDL on the binding of Dil-labeled OxLDL to CHO-CD36 cells**. CHO-CD36 cells were first incubated with the anti-CD36 antibody FA6 (panel A) or mouse serum IgG (Panel B) or unlabeled oxLDL (panel C) and then 10 μg/ml of Dil-labeled OxLDL was added to incubate for a further 2 – 4 hours. The concentrations of anti-CD36 antibody in panel A and of mouse IgG serum in panel B from top to bottom are: 0, 2, 4, 6, 8 and 10 μg/ml respectively. The concentrations of unlabelled OxLDL in panel C from top to bottom are: 20, 40, 100, 200, and 400 μg/ml. Lipoprotein binding to cells (expressed as mean relative fluoresence) was determined by flow cytometry.

Previous reports have indicated that CD36 expressed in Sf 9 and COS 7 cells [24] interacts with OxLDL, LDL and HDL, while CD36 expressed in CHO cells does not [35]. To determine if cell type specific lipoprotein binding was present in different cell lines, Sf9-CD36, CHO-CD36 and C32 were incubated with DiI labeled lipoprotein (1,1'diotadecyl3-3'-3'-tetramethylindocarbo-cyanine perchlorate (Dil) [36] prior to examination by fluorescence microscopy. Sf9 cells infected with baculovirus containing the gene for human CD36 displayed an intense staining with each of the three lipoproteins (Fig. 1, Sf9-CD36), as has been previously reported [24,33]. Whereas mock-transfected Sf9 cells did not bind DiI-labeled lipoproteins (Fig. 1, Sf9). CHO cells that expressed the CD36 gene bound DiI-oxLDL but not Dil-HDL or Dil-LDL. (Fig. 1, CHO-CD36). CHO-mock cells did not interact with any of the lipoproteins (Fig. 1, CHO-mock). These ligand preferences were consistent with our previous observations, however we wished to extend these observations to human cells. We therefore repeated our experiments using C32 amelanotic melanoma cells, which endogenously express human CD36 [37-39]. The ligand preferences of C32 cells were found to be similar to CHO-CD36 cells (Fig. 1, C32). The interaction was specific to CD36 since receptor blockade with an anti-CD36 antibody (Fig. 2A) and competition with unlabeled oxLDL (Fig. 2C) inhibited the interaction of labeled oxLDL with CHO-CD36 cells.

### Effect of filipin on lipoprotein binding to CHO-CD36 and C32 cells

**Figure 3:**
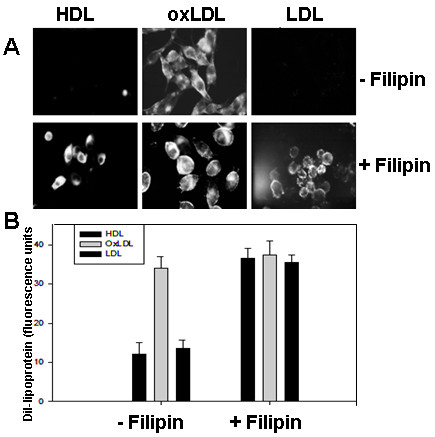
**Effects of filipin on Dil-lipoprotein binding to CHO-CD36 cells**. Panel A: CHO-CD36 cells were pre-treated with 10 μg/ml of filipin at 37°C for 30 minutes and then they were exposed to 10 μg/ml of Dil-lipoprotein at 37°C for 4 hours. The cells were fixed with 4% formaldehyde and viewed under fluorescent microscope. Panel B: Cells were treated same as in A and were then assayed for the presence of Dil by flow cytometry. The data in B represents an average result of three independent experiments.

**Figure 4:**
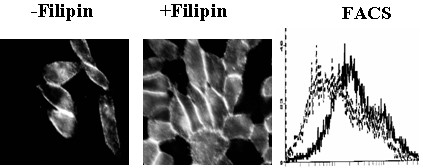
**Effects of filipin on CD36 surface protein expression in CHO-CD36 cells**. CHO-CD36 cells were treated with filipin at 10 μg/ml and assayed for CD36 protein expression by immuostaining with FITC-conjugated anti-CD36 antibody (Left panel, no filipin treated; middle panel, filipin treated showing higher fluoreence indensity) and flow cytometry (right panel, dark traces treated with filipin and gray traces without filipin treatment as control).

CD36 has palmitoylated cysteine residues in its transmembrane regions which is consistent with its localization to caveolae [40-42]. Numerous receptors are known to change their behavior when they become associated with caveolae [29,43,44], therefore we examined the effect of the caveolae disrupting agent filipin [45] on the interaction between CHO-CD36 cells and DiI-lipoproteins. Untreated CHO-CD36 cells bound only oxLDL (Fig. 1), however when CHO-CD36 cells were exposed to filipin at 10 μg/ml for 30 min, they bound all three Dil-lipoproteins (Fig. 3A). Compared to untreated samples, filipin increased CHO-CD36 cell-associated Dil-HDL and Dil-LDL by 160% and 120% respectively, however no significant change was detected for DiI-oxLDL binding to CHO-CD36 cells (Fig. 3B). C32 cells, which natively express CD36, were treated in the same manner and results similar to those seen in CHO-CD36 cells were obtained (results not shown). Caveolae are surface invaginations or clefts [46]. To determine if the observed binding changes were accompanied by an increase or decrease in surface accessible CD36, we evaluated CD36 surface expression by flow cytometery using an anti-CD36-FITC conjugated antibody. Upon treatment with filipin CD36 detectable on the surface was significant increased in CHO-CD36 (Fig. 4).

### Changes in CD36 ligand preference are associated with disruption of caveolae-enriched membrane fraction

**Figure 5:**
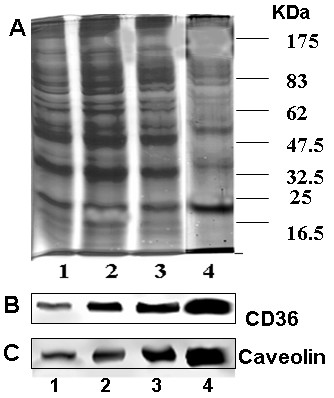
**Purification of caveolin-enriched membrane fraction from CHO-CD36 cells and identification the colocalization of caveolin and CD36 proteins**. Purification of the caveolin-enriched membrane fraction was as described in Materials and Methods. A: Coomassie blue staining of protein profile from purification fractions. Lane 1, whole cell lysate (80 μg); lane 2, post nuclear supernatant (60 μg), lane 3, Optiprep gradient fraction (30 μg), and lane 4, caveolin-enriched membrane fraction (20 μg). The same amount of proteins (30 μg) from each fraction as in Panel A was analyzed with Western blots by anti-CD36 antibody (B) and anti-caveolin antibody(C).

**Figure 6:**
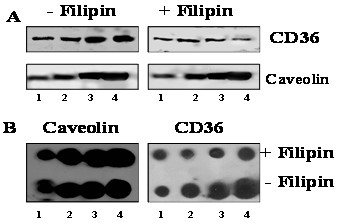
**Semi-quantitation of caveolin and CD36 with and without filipin treatment**. Caveolin-enriched membrane protein fractions same as in Fig. 5 were analyzed with both anti-CD36 antibody (upper panel in A) and anti-caveolin antibody (lower panel in A) for the two protein presence. B. Quantitative comparison of caveolin and CD36 proteins in final caveolin-enriched membrane fraction from either filipin treated or untreated cells with dot blots. From left to right, 5, 10, 20 and 40 μg/dot of total proteins were loaded.

**Figure 7:**
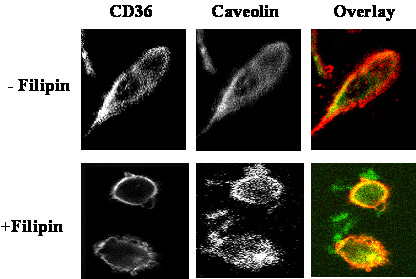
**Redistribution of caveolin and CD36 proteins on CHO-CD36 upon filipin treatment**. CHO-CD36 cells upon treated with filipin and then fixed with 4% formaldehyde in PBS, and double immunostained with either FITC-conjugated anti-CD36 antibody and rodomin-conjugated anti-caveolin antibody. The stainned cells were examined under confocal microscope. Upper panel: no filipin was treated and lower panel: filinpin was treated showing more CD36 staing in cytoplam. Magnifications 400×.

To confirm the hypothesis that filipin treatment causes CD36 to dissociate from caveolae-enriched membrane in CHO CD36, we partially purified the caveolin-enriched membrane fraction from CHO-CD36 cell lysate using a detergent-free method [47]. Immunoblotting of the fractions obtained from untreated CHO-CD36 cells with both an anti-CD36 antibody and an anti-caveolin antibody indicated that CD36 was present in the caveolin-enriched membrane faction (Fig. 5). In contrast, when cells were treated with filipin the amount of CD36 in the final two fractionation steps was greatly diminished (lane 3, 4, Fig. 6A). A semi-quantitative comparison of the caveolin-rich membrane fractions from filipin-treated and untreated CHO-CD36 cells indicated that the amount of CD36 in the final purification fractions of filipin treated cells was decreased by about 30 – 40% relative to the untreated samples (Fig 6B). No difference was seen for the amount of caveolin protein present in the filipin-treated and untreated fractions (Fig. 6B) suggesting that the redistribution of CD36 and its change in ligand preference are related events. Double immunoflorescent staining of CHO-CD36 cells with both anti-CD36 and anti-caveolin antibodies showed that cells without filipin treatment, both CD36 and caveolin concentrated around membranes (Fig. 7,-Filipin), and however, when treated with filipin, more CD36 proteins appeared in the cytoplasm (Fig. 7, +Filipin).

## Discussion

We conclude that the local plasma membrane environment in which CD36 is located, e.g. whether CD36 is present in caveolae or in the general membrane fraction, influences the receptor's capacity to bind to native lipoproteins. This conclusion may reconcile why CD36 was originally identified as a receptor for oxLDL, but not native HDL or LDL or acetyl LDL [48,49] when CD36 was expressed in 293 cells, but that recent studies using CD36 transfected Sf9 and COS-7 cells have indicated that native HDL, LDL, VLDL, as well as OxLDL all bind with high affinity to CD36 [24,33]. It is also consistent with the observation that the interaction of *Plasmodium falciparum*-infected erythrocytes with CD36 is inhibited by some ligands (i.e. pRBCs and oxLDL) in all cell types, while inhibition with other ligands (i.e. LDL and HDL) were dependent on the expression system used [33].

Our initial experiments confirmed and extended the binding studies of Calvo et al [8,24] and focused on ensuring that the ligand preference of CD36 expressed in Sf9 cells, CHO cells and C32 cells was, as we and others, have described it (Fig. 1). We are confident that the majority of the lipoprotein binding observed with Sf9 and CHO cells is due to the CD36 receptor that has been introduced into these cell lines since mock (either baculovirus control or vector only) cell line controls show minimal amounts of lipoprotein binding. A similar control cell line is not available for C32 cells, however: 1) the staining pattern of these cells strongly resembles that seen in CHO-CD36 cells; and 2) C32 cells support pRBC adherence in the presence of native lipoproteins, which suggests that unoxidized lipoproteins do not compete for CD36 in this system. We therefore conclude that C32 cells may reflect the ligand preferences of CD36 in vivo.

We have previously speculated that expressing human CD36 in several different cell types could lead to a common amino acid sequence but with cell type specific gylcosylation states or covalent modifications and that these may determine ligand preference. While we cannot rule out this possibility, the effect of filipin, which modifies the properties of the lipid membrane [44], strongly suggests that differences in native lipoprotein ligand preference do not result from covalent modification alone. Similarly, the relatively short time required to alter the ligand preference of the CHO CD36 and C32 cells suggests that alterations of the amount or structure of the receptor because of altered synthesis rates [27] are not involved. Filipin is able to bind to and remove cholesterol from mammalian membranes, particularly from the cholesterol enriched segments that form invaginated regions of the membrane responsible for endocytosis, mechanotransduction, signaling, cholesterol exchange [50] and the induction of apoptosis [50].

While our results confirmed that CD36 was colocalized with the protein marker of caveolae, caveolin-1, which suggested that the entry and exit of CD36 into caveolae may function as a mechanism to control the cholesterol content of a cell. Caveolae are cell surface plasma membrane invaginations observed in different type of cells and their protein marker caveolin-1 has been implicated in the development of an atheroma and involved in regulating several signal transduction pathways and processes that play an important role in atherosclerosis[51,52]. Fielding et al (1997, 1995) have also observed that caveolae are clathrin-free cell-surface organelles implicated in transmembrane transport. When 3H-labeled free cholesterol was selectively transferred to the cells from labeled low density lipoprotein to increase cell free cholesterol approximately 15%, there was a 6-fold increase in label in the caveolar fraction above baseline levels. When okadaic acid was used, it decreased cholesterol efflux, which indicate that caveolae represent a major site of efflux of both newly synthesized and low density lipoprotein-derived free cholesterol in cells[53,54]. In our study, the lipoprotein binging profile of in CHO-CD36 cells was altered by filipin treatment (Fig. 3) and the filipin treatment could alter the redistribution of CD36 molecules in caveolae into cytoplasm.Gaus et al.(2005) demonstrated that the caveolae proteins in the depleted membrane are affected differently by detergents and the depletion of chlosterol severely alters lipid raft structure, causing the dipersal of caveolar raft-assiciated proteins into non- raft domains of the plasma membrane [55] However, its molecular mechanism how CD36 and caveolin interaction in regulating lipoprotein transport remains to be further studied.

Filipin treatment of CHO-CD36 cells appeared to have little effect on the capacity of CD36 to interact with oxLDL (Fig. 3) or pRBC (data not shown). This may appear to contradict the findings of Frank et al [51] that treatment of macrophages with cyclodextran reduces oxLDL uptake, however these results were obtained after a much longer treatment period (16 hr) and were found to be due to the regulation of CD36 expression. Short term treatments produced a translocation of CD36 accompanied by a change in the surface distribution of CD36 and an increase in the staining intensity [56]. Similar results were obtained with C32 cells and therefore we expect that sequestration of CD36 in caveolae may be a physiologically relevant short term mechanism of lipid traffic control. Further studies are needed to determine the specific binding sites of CD36 on caveolin molecules and their signal regulation pathways, which may provide a pathological implication in lipid-related diseases.

## Abbreviations

HDL: High Density Lipoprotein; HEPES: 4-(2-Hydroxyethyl)-1-Piperazineethane Sulfonic acid; LDL: Low Densivity Lipoprotein; MARCO: Microphage Receptor with Collagenous Domain; oxLDL: oxidized LDL; PRBCs: Plasmodium falciparum-infected lood cells; SR-B1: Scavenger Receptor B1; VLDL: Very Low Density Lipoprotein.

## Authors' contributions

JZ performed all of the experiements and data analysis, and drafted the manuscript, WC carried out data analysis, figure formatting and manuscript proofreading, IC conceived of the study, and participated in its design and experimental instruction and the manuscript preparation. All authors read and approved the final manuscript.
